# COVID-19 vaccine boosters for young adults: a risk benefit assessment and ethical analysis of mandate policies at universities

**DOI:** 10.1136/jme-2022-108449

**Published:** 2022-12-05

**Authors:** Kevin Bardosh, Allison Krug, Euzebiusz Jamrozik, Trudo Lemmens, Salmaan Keshavjee, Vinay Prasad, Marty A Makary, Stefan Baral, Tracy Beth Høeg

**Affiliations:** 1 School of Public Health, University of Washington, Seattle, Washington, USA; 2 Edinburgh Medical School, University of Edinburgh, Edinburgh, UK; 3 Epidemiology, Artemis Biomedical Communications, Virginia Beach, Virginia, USA; 4 University of Oxford Wellcome Centre for Ethics and Humanities, Oxford, UK; 5 Faculty of Law and Dalla Lana School of Public Health, University of Toronto, Toronto, Ontario, Canada; 6 Department of Global Health and Social Medicine, Harvard Medical School, Boston, Massachusetts, USA; 7 Epidemiology and Biostatistics, University of California San Francisco, San Francisco, California, USA; 8 Department of Surgery, Johns Hopkins University, Baltimore, Maryland, USA; 9 Epidemiology, Johns Hopkins University Bloomberg School of Public Health, Baltimore, Maryland, USA; 10 Clinical Research, Acumen, LLC, Burlingame, California, USA; 11 Sierra Nevada Memorial Hospital, Grass Valley, California, USA

**Keywords:** COVID-19, Epidemiology, Ethics- Medical, Civil Rights, Coercion

## Abstract

In 2022, students at North American universities with third-dose COVID-19 vaccine mandates risk disenrolment if unvaccinated. To assess the appropriateness of booster mandates in this age group, we combine empirical risk-benefit assessment and ethical analysis. To prevent one COVID-19 hospitalisation over a 6-month period, we estimate that 31 207–42 836 young adults aged 18–29 years must receive a third mRNA vaccine. Booster mandates in young adults are expected to cause a net harm: per COVID-19 hospitalisation prevented, we anticipate at least 18.5 serious adverse events from mRNA vaccines, including 1.5–4.6 booster-associated myopericarditis cases in males (typically requiring hospitalisation). We also anticipate 1430–4626 cases of grade ≥3 reactogenicity interfering with daily activities (although typically not requiring hospitalisation). University booster mandates are unethical because they: (1) are not based on an updated (Omicron era) stratified risk-benefit assessment for this age group; (2) may result in a net harm to healthy young adults; (3) are not proportionate: expected harms are not outweighed by public health benefits given modest and transient effectiveness of vaccines against transmission; (4) violate the reciprocity principle because serious vaccine-related harms are not reliably compensated due to gaps in vaccine injury schemes; and (5) may result in wider social harms. We consider counterarguments including efforts to increase safety on campus but find these are fraught with limitations and little scientific support. Finally, we discuss the policy relevance of our analysis for primary series COVID-19 vaccine mandates.

## Introduction

COVID-19 vaccine booster mandates have been controversial, especially in younger age groups. Two main factors continue to drive scientific controversy: a lack of evidence that booster doses provide a meaningful reduction in hospitalisation risk among healthy adolescents and young adults, and mounting evidence that widespread prior infection confers significant protection against hospitalisation due to (re)infection. Further, mandates have deleterious societal consequences and are eroding trust in scientific and government institutions.^1^ In North America, as of May 2022 at least 1000 colleges and university campuses required COVID-19 vaccination, and over 300 required boosters.^2^ More than 50 petitions have been written opposing these vaccine mandates,^3^ raising specific legal and ethical complaints.^4^ To our knowledge, few have changed their vaccine guidance for the 2022–2023 academic year and several have mandated the new bivalent booster.

Policymakers, public health scholars and bioethicists have argued both for and against COVID-19 vaccine mandates. The strongest argument made by proponents of vaccine mandates is based on the harm principle: insofar as vaccines prevent transmission and thereby reduce harm to others, restrictions on individual freedom are viewed as more ethically justifiable.^5^ However, a reduction in risk to others (especially if this is a small or temporary effect) might not alone be sufficient to justify a booster mandate in young people. Savulescu^6^ and Giubilini and colleagues^7^ have argued that, to be ethical, vaccine mandates require four conditions: that the disease be a grave public health threat; that there is a safe and effective vaccine; that mandatory vaccination has a superior cost/benefit profile in comparison to other alternatives; and that the level of coercion is proportionate.

Proportionality is a key principle in public health ethics.^1^ To be proportionate, a policy must be expected to produce public health benefits that outweigh relevant harms, including harms related to coercion, undue pressure, loss of employment and education and other forms of liberty restriction. Williams^8^ has argued that COVID-19 vaccine mandates may be justified for older but not younger people, among whom such policies are not proportionate given a lack of clarity that benefits outweigh harms. Such ethical assessments should rely on empirical data: thorough risk-benefit assessment requires quantification (where possible) of relevant risks and benefits *for the group affected by the policy*. With respect to poor outcomes due to COVID-19, the most consistent predictors are age^9^ and comorbidities.^10^ Similarly, age and sex are prominent risk factors for vaccine-associated reactogenicity^11^ and serious adverse events (SAE) such as myocarditis, which is more common in young males.^12^ Vaccine requirements should therefore be predicated on an age-stratified and sex-stratified risk-benefit analysis and consider the protective effects of prior infection.^13^

In this paper, we integrate a risk-benefit assessment of SARS-CoV-2 boosters for adults under 30 years old with an ethical analysis of mandates at universities. Our estimate suggests an expected net *harm* from boosters in this young adult age group, whereby the negative outcomes of all SAEs and hospitalisations may on average outweigh the expected benefits in terms of COVID-19 hospitalisations averted. We also examine the specific harms to males from myo/pericarditis. We then outline a five-part ethical argument empirically assessing booster mandates for young people informed by the quantitative assessment. First, we argue that there has been a lack of transparent risk-benefit assessment; second, that vaccine mandates may result in a net expected harm to individual young adults; third, that vaccine mandates are not proportionate; fourth, that US mandates violate the reciprocity principle because of current gaps in vaccine injury compensation schemes; fifth, that mandates are even less proportionate than the foregoing analyses suggest because current high levels of coercion or pressure may create wider societal harms. We consider possible counterarguments including potential rationales for mandates based on a desire for social cohesion or safety and summarise why such arguments cannot justify current COVID-19 vaccine mandates. We suggest that general mandates for young people ignore key data, entail wider social harms and/or abuses of power and are arguably undermining rather than contributing to social trust and solidarity.

## Background

To provide background for our risk-benefit assessment and ethical arguments, we outline recent controversies among experts regarding vaccine boosters and summarise current data on COVID-19 vaccines, specifically: vaccine effectiveness against transmission, effectiveness in those with prior infection and the age-stratified risk of severe COVID-19.

### Controversy among experts

Most countries outside of North America have not required or mandated booster doses for young healthy adults at universities,^14^ suggesting that, at a minimum, there is a diversity of expert views on whether the expected benefits of such policies outweigh their potential harms. In July 2021, the Centers for Disease Control and Prevention (CDC) released a joint statement with the Food and Drug Administration (FDA)^15^ reassuring the public that boosters were not necessary. Just 2 months later, in September 2021, a US FDA advisory committee overwhelmingly voted 16-2 against boosting healthy young adults.^16^ Yet, this recommendation was over-ruled by the White House and CDC leading to the resignation of two high-level FDA vaccine experts. These experts wrote in *The Lancet* about the ‘…need to identify specific circumstances in which the direct and indirect benefits of doing so are, on balance, clearly beneficial’.^17^ To date, the only risk-benefit assessment made public has narrowly focused on myo/pericarditis in the absence of sufficient safety data from an appropriately powered trial.^18^ In fact, the CDC’s own evidence-to-risk framework found no COVID-19 hospitalisation in either booster (three-dose) or placebo (two-dose) groups of the BNT162b2 booster trial.^19^

Because the mRNA vaccine third-dose booster trials were too small to measure important clinical endpoints, additional doses have been granted Emergency Use Authorization (EUA) based on observational data suggesting benefits in older populations.^19^ Prior to the emergence of the Omicron variant, the US CDC estimated^19^ that administering a booster dose to 8738 (BNT162b2) or 11 994 (mRNA-1273) 18–29 year-olds would prevent one COVID-19 hospitalisation over 6 months. As of August 2022, this estimate had not been updated to reflect increasing natural immunity or waning vaccine effectiveness. Data on booster vaccine effectiveness specific to young adults are scarce; reports typically either do not provide stratified data below a certain age (eg, 50 years^20^) or use younger adults as the baseline to assess effectiveness in older adults (in part because severe disease is already extremely rare in non-boosted young adults).^21^ In a recent CDC publication, which stratified for ages 18–49, a booster dose increased effectiveness against emergency department encounters and hospitalisations among immunocompetent adults during the Omicron wave, but the analysis did not adjust for comorbidities and excluded those with a history of prior infection ‘to reduce the influence of protection from previous infection’.^22^

Risk-benefit calculations for the primary series among younger children and adolescents are similarly limited. A cohort study conducted in Hong Kong estimated the number needed to harm (NNH) from myo/pericarditis for dose 2 of BNT162b2 was 2563 among adolescent males,^23^ yet there was no US-specific NNH published by the CDC, nor did the agency recommend shifting to a one-dose policy for adolescents as did the UK, Norway, Taiwan and Hong Kong.^23^ The CDC first presented a risk-benefit analysis of booster vaccination in September 2021, yet the harms focused strictly on myocarditis versus all SAEs and collapsed age strata with very disparate myocarditis risks.^24^ Moreover, the CDC’s outdated risk-benefit analysis for adolescents and young adults does not distinguish important subgroups such as or those who have recovered from previous infection or healthy young people (as opposed to those with comorbidities or immunocompromised status).^24^

### Current data regarding COVID-19 vaccines

A thorough ethical evaluation of risks and benefits requires relevant empirical data, especially where risks and benefits can be quantified to a reasonable degree of certainty. Relevant data include those regarding average individual vaccine safety and effectiveness and age stratification of these data as well as the protective effect of prior infection and the effectiveness of vaccines against transmission.

Proponents of mandates have argued that current vaccines prevent transmission, which would support a standard ethical reason in favour of mandates: the protection of others. Yet it is increasingly clear that current vaccines provide, at most, partial and transient protection against infection, which decreases precipitously after a few months,^25 26^ with limited effects on secondary transmission.^27 28^ The CDC states: ‘anyone with Omicron infection, regardless of vaccination status or whether or not they have symptoms, can spread the virus to others.’^29^ It is therefore inaccurate in 2022 to infer a sustained or long-term reduction in transmission from a short-term reduction in infection.^30^

A second limitation is ignoring the protective effects of prior infection. In February 2022, the CDC estimated that 63.7% of adults aged 18–49 years had infection-induced SARS-CoV-2 antibodies, up from 30% in September 2021.^13^ By September 2022, the majority of young adults, both vaccinated and unvaccinated, are estimated to have been previously infected with COVID-19. Evidence increasingly shows that prior SARS-CoV-2 infection provides at least similar (and perhaps more durable) clinical protection to current vaccines,^31–33^ which current university policies fail to acknowledge (in addition to more general uncertainties about risks and benefits in relevant age groups^34^).

Mass vaccination had been proposed as a way to ‘end the pandemic’.^35^ However, elimination or eradication of the virus is not a tenable goal with vaccines that provide only temporary and incomplete reduction in infection risk, and the presence of multiple animal reservoirs. Because of this, nearly all human beings will eventually be infected with SARS-CoV-2, as with other endemic coronaviruses (and every pandemic influenza virus on record), many times in their life.^36^ Denmark has, for example, acknowledged vaccinating children was not effective at curbing spread of the virus and is no longer recommending vaccination against COVID-19 for most children.^37 38^ Taking population immunity into account with variant severity and projected coincident surges of influenza, SARS-CoV-2 and respiratory syncytial virus in the winter of 2022–2023, the UK’s Joint Committee on Vaccination and Immunisation (JCVI) currently recommends that high-risk groups be *offered* a booster.^39^

A fourth point relates to the burden of COVID-19 in young adults under 40. Using pre-COVID-19-vaccinera mortality data from 190 countries, the adjusted infection fatality ratio for 18–29 year-olds ranged from 100 per million (18 year-olds) to 500 per million (29 year-olds) with significant variation by country within each age stratum.^40^ A recent study from South Africa during the Omicron BA.1–BA.4/5 wave demonstrates that despite a high proportion of breakthrough infections, the risk of hospitalisation remains lowest among young adults.^41^

While both vaccination and prior infection can substantially reduce the likelihood of COVID-19 mortality,^32 33 39^ the protection against hospitalisation afforded by a booster wanes rapidly.^41^ The study from South Africa demonstrated that protection waned to less than 50% after 3–4 months.^41^ Protection against symptomatic disease can be initially restored but wanes approximately 10 weeks after a booster dose^42^; in the study from England, protection against severe disease could not be measured with the test-negative case–control design due to the few cases of severe disease during Omicron.^42^ Using a national population-wide data set in Qatar, both previous infection alone and vaccination alone were found to provide >70% protection against severe Omicron (BA.1 or BA.2) disease.^43^ However, the stratified data in Altarawneh, et al. supplemental table S5 show that prior infection alone was 91% effective against severe Omicron disease, whereas protection from two or three doses of vaccine alone was 66% and 83%, respectively.^43^

Finally, COVID-19 does cause acute illness, and may have long-term effects (Long COVID) for some, particularly those who develop critical illness, but vaccination may not entirely prevent longer term sequelae^44^ and the existing data are non-randomised, from variants that predate Omicron and with unclear relevance for adults under age 40. The existence of effective treatments for clinical management^45^ is also an argument against vaccine mandates, especially for groups not considered at risk for severe illness.

## Risk-benefit assessment

**Figure 1:**
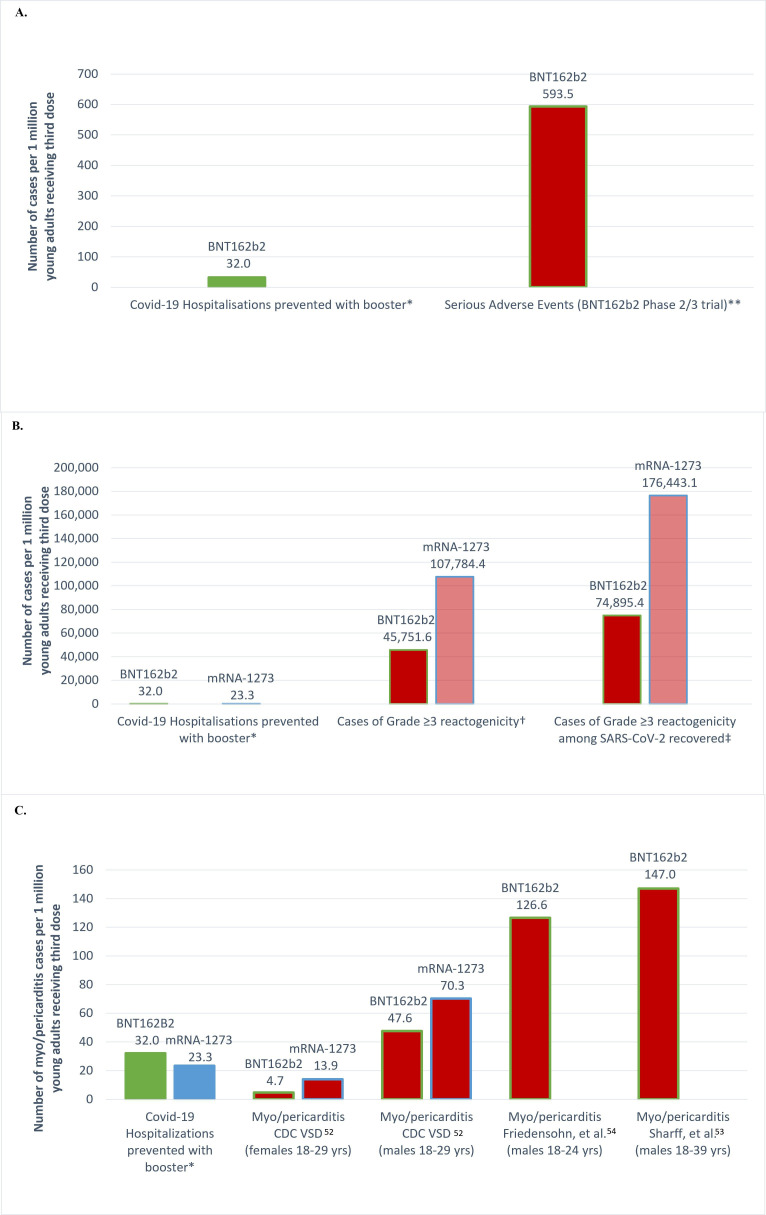
(A, B, C) Expected hospitalisations prevented over six months and serious adverse events (SAEs), cases of grade ≥3 reactogenicity, and vaccine-associated myo/pericarditis among 18–29-year-olds per million BNT162b2 and mRNA-1273 booster vaccinations. *CDC-estimated number needed to vaccinate (NNV) with a booster to prevent 1 hospitalisation over 6 months in 18–29-year-olds^18^ was adjusted for reduced Omicron severity (aOR=0.28)^47^ as follows: BNT162b2 (8738/0.28=31 207) and mRNA-1273 (11 994/0.28=42 836). Per million third doses, hospitalisations prevented for BNT162b2 were computed as follows: 1/(8738/0.28)×10^6^=1/31 207×10^6^=32.0 and 1/(11 994/0.28)×10^6^=1/42 836×10^6^=23.3 for mRNA-1273 **SAEs: Three serious adverse events among BNT162b2 booster recipients were deemed by blinded investigators to be related to vaccination (3/5055). These included: moderate persistent tachycardia, moderate transient elevated hepatic enzymes, and mild elevated hepatic enzymes.^18 50^ †Reactogenicity rates are BNT162b2 (14/306) and 45 751.6 per million third doses; mRNA-1273 (18/167) and 107 784.4 per million third doses.^50^ ‡Estimated reactogenicity rates were computed assuming 63.7% seroprevalence^13^ and at least 2x reactogenicity among those with prior SARS-CoV-2 infection.^56 57^

In a recent editorial, vaccine developer and paediatrician Paul Offit^34^ argued: ‘because boosters are not risk-free, we need to clarify which groups most benefit.’^1^ Below, we provide an Omicron-specific risk-benefit assessment of booster vaccination for young adults aged 18–29 years for both Pfizer (BNT162b2) and Moderna (mRNA-1273) vaccines. This analysis builds on a stratified risk-benefit analysis of vaccination among adolescents aged 12–17 years.^46^ For the booster among young adults aged 18–29 years, the calculations use the CDC’s pre-Omicron number needed to vaccinate (NNV),^19^ the estimated reduction in severity of Omicron versus Delta^47^ and current estimated seroprevalence.^13^ While harms from COVID-19 vaccines are uncommon,^48^ they should be factored into policy recommendations. This risk-benefit analysis considers the overall rate of reported SAEs (figure 1A) and grade ≥3 reactogenicity (figure 1B) and myo/pericarditis among males (figure 1C). Rates and definitions are consolidated in table 1A,B,C.

**Table 1:** Risk-benefit analysis of third mRNA vaccination: definitions and rates for serious adverse events (SAEs), grade ≥3 reactogenicity and myo/pericarditis in 18–29 year-olds by manufacturer

1A. Serious adverse events (SAE) and risk-benefit analysis	Rate	Risk	Harms per 1 million third doses	Hospitalisations prevented per 1 million third doses Absolute risk reduction=(1/adj NNV)×10^6^	Risk-benefit ratio of third-dose SAEs per COVID-19 hospitalisation prevented
**SAE** An adverse event that results in any of the following conditions: death, life threatening at the time of the event, inpatient hospitalisation or prolongation of existing hospitalisation; persistent or significant disability/incapacity, a congenital anomaly/birth defect or a medically important event, based on medical judgement.	BNT162b2 3/5055^19^ Slide 26	1 in 1685	593.5 (10^6^/1685)	BNT162b2^19 47^ 1/(8738/0.28)×10^6^=1/31 207×10^6^=32.0 hospitalisations prevented per million third doses	18.5/1 593.5/32.0=18.5 (BNT162b2 SAE^19^/BNT162b2 hospitalisations prevented)
	mRNA-1273 0/171^50^ Table 4b	Not calculable*	Not calculable*	mRNA-1273^19 47^ 1/(11 994/0.28)×10^6^=1/42 836×10^6^=23.3 hospitalisations prevented per million third doses	Not calculable*

1B. Grade ≥3 reactogenicity and risk-benefit analysis	Rate	Risk	Harms per 1 million third doses	Hospitalisations prevented per 1 million third doses (as above)	Risk-benefit ratio of third-dose grade ≥3 reactogenicity per COVID-19 hospitalisation prevented
**Grade ≥3 reactogenicity** Defined as local/systemic adverse events that prevent daily routine activity or require use of a pain reliever (grade 3) or require an emergency room visit or hospitalisation (grade 4).	BNT162b2 14/306^50^ Table 3f	1 in 22	45 751.6 (14/306)×10^6^	BNT162b2: 32.0	1429.7/1 (45 751.6/32.0)
	mRNA-1273 18/167^50^ Table 3f, 4b	1 in 9	107 784.4 (18/167)×10^6^	mRNA-1273: 23.3	4625.9/1 (107 784.4/23.3)
	**2× reactogenicity among SARS-CoV-2 recovered**				
	BNT162b2^50 56^	1 in 11	74 895.4 0.637*2*(14/306)×10^6^+0.363*(14/306)×10^6^	BNT162b2: 32.0	2340.5/1 (74 895.4/32.0)
	mRNA-1273^50 56^	1 in 4.5	176 443.1 0.637*2*(18/167)×10^6^+0.363*(18/167)×10^6^	mRNA-1273: 23.3	7572.7/1 (176 443.1/23.3)

1C. Myo/pericarditis and risk-benefit analysis	Harms per 1 million third doses (males)	Harms per 1 million third doses (females)	Hospitalisations prevented per 1 million third doses (as above)	Risk-benefit ratio of third-dose myo/pericarditis per COVID-19 hospitalisations prevented	
**Myocarditis** Presence of ≥1 new or worsening‡: Chest pain/pressure/discomfort Dyspnoea/shortness of breath/pain with breathing Palpitations Syncope AND ≥1 new finding of: Troponin above normal limit Abnormal ECG, EKG or rhythm monitoring findings consistent with myocarditis Abnormal cardiac function or wall motion abnormalities on ECHO cMRI findings consistent with myocarditis AND No other identifiable cause of symptoms **Pericarditis** Presence of ≥2 new or worsening: Acute chest pain§ Pericardial rub on exam New ST-elevation or PR-depression on EKG New or worsening pericardial effusion on echocardiogram or MRI	**Ages 18–39**	**Ages 18–39**		**Males**	**Females**
	BNT162b2 147/million^53^ (Sharff *et al*)	n/a	BNT162b2: 32.0 mRNA-1273: 23.3	BNT162b2 4.6/1 (147.0/32.0)	n/a
	**Ages 18–29**	**Ages 18–29**			
	BNT162b2 (VSD): 47.6/million^52^ Slide 23	BNT162b2 (VSD): 4.7/million^52^ Slide 23		BNT162b2 1.5/1 (47.6/32.0)	BNT162b2 0.2/1 (4.7/32.0)
	mRNA-1273 (VSD): 70.3/million^52^ Slide 23	mRNA-1273 (VSD): 13.9/million^52^ Slide 23		mRNA-1273 3.0/1 (70.3/23.3)	mRNA-1273 0.6/1 (13.9/23.3)
	**Ages 18–24**	**Ages 18–24**			
	BNT162b2 126.6/million^54^ (Friedensohn et al**)	n/a		BNT162b2 3.9/1 (126.6/32.0)	n/a
	**Ages 16–17**†	**Ages 16–17**†			
	BNT162b2 (VSD): 200.3/million^51^ Slide 25	BNT162b2 (VSD): 44.0/million^51^ Slide 25		BNT162b2 6.3/1 (200.3/32.0)	BNT162b2 1.4/1 (44.0/32.0)

*Footnote (h) from GRADE Table 3e: Overall, 4/344 (1.2%) participants experienced five SAEs during a median follow-up of 5.7 months after booster dose (administered at least 6 months after a 50 μg (n=173) or 100 μg (n=171) two-dose primary series); the sponsor deemed these unrelated to mRNA-1273. Data on an equivalent primary series comparison group were not available at the time of the GRADE assessment.^50^

**Based on hospitalised cases only within 21 days of receipt of mRNA-1273.^54^

†COVID-19 hospitalisation risk for aged 16–17 years is lower than for those aged 18–29 years, thus the risk/benefit ratio provided is an underestimate.

‡Criteria for probable case. Confirmed case requires symptoms plus histopathological evidence OR elevated troponin AND cMRI findings.

§Typically described as pain made worse by lying down, deep inspiration or cough and relieved by sitting up or leaning forward, although other types of chest pain may occur.

cMRI, cardiac MRI; GRADE, Grading of Recommendations, Assessment, Development, and Evaluation; NNV, number needed to vaccinate; VSD, Vaccine Safety Datalink.

SAEs^49^ include those that: result in death or are life threatening; result in hospitalisation, prolongation of hospitalisation or significant disability/incapacity; cause a congenital anomaly/birth defect; or cause other medically important events.^2^ Grade 3 or 4 reactogenicity is defined as local/systemic events that prevent daily routine activities or require use of a pain reliever (grade 3) or resulting in an emergency room visit or hospitalisation (grade 4).^49 50^

To estimate the expected harms (SAEs including myo/pericarditis and grade ≥3 reactogenicity) and benefits (COVID-19 hospitalisations prevented) specific to boosting young adults aged 18–29 years, we used data reported by CDC from phase II/III clinical trials,^19 50–52^ peer-reviewed observational data from large integrated health systems^53–57^ and postmarketing surveillance collected via V-Safe by the CDC.^58^ We compute harms and benefits per single hospitalisation averted as well as per million third doses administered.

### Hospitalisations prevented

To estimate the benefits of hospitalisations prevented by boosters, we updated the CDC’s estimated NNV^19^ for Omicron, which was found to be markedly less virulent than Delta.^47^ We selected Trobajo-Sanmartín *et al* because the analysis provides stepwise comparisons of Omicron BA.1 to Delta (adjusted OR (aOR)=0.28, 95% CI 0.16 to 0.47), as well as the more recent BA.2 to BA.1 (aOR=0.52, 95% CI 0.29 to 0.95). To be conservative, we used the BA.1 versus Delta aOR rather than attempting to estimate the combined BA.2 versus Delta risk reduction. Scaling the CDC’s NNV estimates of 8738 for BNT162b2 and 11 994 for mRNA-1273 by this reduced severity, we estimate that 31 207 (8738/0.28) to 42 836 (11 994/0.28) young adults would need to be boosted with BNT162b2 or mRNA-1273, respectively, to prevent one COVID-19 hospitalisation over a 6-month period. Hospitalisations prevented per million BNT162b2 and mRNA-1273 doses administered are 32.0 and 23.3, respectively (table 1).

### SAE rates reported from manufacturer-provided data

Of the 12 SAEs reported in the intervention arm of the randomised controlled trial (RCT) for BNT162b2 (n=5055), three were found by blinded investigators to be attributable to the vaccine, providing a rate of 1 in 1685 (3/5055).^19^ The three SAEs considered vaccine related included: moderate persistent tachycardia, moderate transient elevated hepatic enzymes and mild elevated hepatic enzymes.^19^ Based on 31 207 in this age group needing to receive the first BNT162b2 booster to prevent one hospitalisation over a 6-month period, the expected SAE rate is 18.5 (3/5055*31 207). (Table 1A) Per million doses administered, the SAE rate is 593.5. Although the safety populations were small, we also reviewed SAEs reported from these cohorts. Pfizer reported 1/306 but the event was not considered related to the vaccine (1/306=0.3%). Similarly, Moderna found that none of the five SAEs experienced by 4 of 344 participants^50^ in its safety population (4/344=1.2%)^3^ were attributable to the vaccine, thus our SAE estimates rely on the only available RCT data (BNT162b2).

### Reactogenicity rates

According to self-report data, side effects from the booster dose prevent on average 28.3% of mRNA vaccine recipients from being able to carry out normal daily activities, typically the day after vaccination.^55^ Sponsor-reported rates from the safety studies for grade ≥3 reactogenicity are 1 in 22 (14/306)^50^ for the BNT162b2 booster to 1 in 9 (18/167)^50^ for the mRNA-1273 booster. Per million third doses, reactogenicity rates are therefore 45 751.6–107 784.4, respectively (table 1B). Per COVID-19 hospitalisation prevented over 6 months in adults aged 18–29 years, the expected number of grade ≥3 reactogenicity cases is therefore 1429.7 (45 751.6/32.0) to 4625.9 (107 784.4/23.3), respectively.

In those with a prior SARS-CoV-2 infection, postvaccination symptoms causing missed work or daily activities are reported twofold^56^ to threefold^57^ more often than those without a history of infection, a major concern given that seroprevalence among adults aged 18–49 years is now well above the February 2022 estimate of 63.7%.^13^ Conservatively assuming 63.7% as the proportion with a history of COVID-19 infection, and a twofold increased likelihood of systemic effects, expected grade ≥3 reactogenicity cases per single hospitalisation prevented would be at least 2340.5–7572.7 for BNT162b2 and mRNA-1273 boosters, respectively (table 1B). Even without taking into account prior infection, the proportion reporting to V-Safe being ‘unable to perform daily activities’ was between 20% and 40% depending on booster product, and higher among those receiving a heterologous booster.^58^

### Booster vaccine-associated myocarditis rates in university-age males 18–29 years

The CDC estimated the rate of postbooster myocarditis during days 0–7 following BNT162b2 vaccine administration in males aged 16–17 years to be approximately 1 in 41 500^51^ using passive surveillance through the Vaccine Adverse Event Reporting System (VAERS), and approximately 1 in 5000^51^ using active surveillance with the Vaccine Safety Datalink (VSD). In males aged 18–29 years, the postbooster myocarditis rate for both products combined using VAERS was reported to be 1 in 101 000^52^ (ages 18–24) to 1 in 208 000^52^ (ages 25–29) while the VSD rate was much higher at 1 in 14 200^52^ (mRNA-1273) to 1 in 21 000^52^ (BNT162b2). Two other population-based studies from the USA and Israel in males aged 18–39 years found the rate to be 1 in 7000 (147.0 per million third doses)^53^ to 9000 (126.6 per million third doses).^54^ In both of these studies, BNT162b2 was the vaccine administered prior to diagnosis. For our estimates, and assuming a precautionary stance, we have used active surveillance rates or population-based rates. For males aged 18–29 years we consider the rate 1 in 7000^53^ to be the most reliable because the method relies on CDC definitions and databases.^59^ We also provide a 16–17 year-old rate because academic acceleration allows some older adolscents to attend college along with the freshman cohort, and in some cases students need to be vaccinated before their 18th birthday to enrol or be assigned to housing. For males aged 16–17 years, we use the VSD rate of 1 in 5000.^51^ In table 1C, we provide a range of myopericarditis estimates for consideration.

### Risk-benefit estimates

The figures display benefits and harms per million third doses administered: SAEs (figure 1A), grade ≥3 reactogenicity (figure 1B) and myopericarditis (figure 1C). At this scale, and as shown in figure 1A, boosting young adults with BNT162b2 could cause 18.5 times *more* SAEs per million (593.5) than COVID-19 hospitalisations averted (32.0).

To prevent one hospitalisation over 6 months by boosting 31 207–42 836 students, a large university campus may also expect 1429.7–4625.9 young adults to experience grade ≥3 reactogenicity disrupting daily activities or requiring medical care when vaccinated with a third dose of BNT162b2 or mRNA-1273, respectively. Per million third-doses of mRNA vaccine administered, between 45 751.6 and 107 784.4 cases of grade ≥3 reactogenicity may be created (figure 1B). Given that prior SARS-CoV-2 infection increases the rate of systemic reactions by twofold to threefold,^56 57^ the number of young adults expected to experience disruptions in their school and daily activities is likely to exceed 74 895.4 with BNT162b2 and 176 443.1 with mRNA-1273 (figure 1B).

Per million third doses of mRNA vaccine administered, 23.3–32.0 hospitalisations may be averted while 47.6–147.0 cases of myo/pericarditis may be caused among young males aged 18–29 years (figure 1C). Thus, to prevent a single hospitalisation among young males aged 18–29 years, we estimate between 1.5 and 4.6 occurrences of myo/pericarditis (rates up to 1 in 7000^53^) among males aged 18–29 years (figure 1C). For adolescents aged 16–17 years and using available data from CDC’s VSD,^51^ we expect 6.3 cases of myo/pericarditis among males and 1.4 among females. Thus, per single hospitalisation averted by boosting 31 207–42 836 young males in this age group, approximately 1.5–6.3 cases of myopericarditis may result.

Most media reports, as well as a recent systematic review^60^ and expert opinion from the American College of Cardiology (ACC),^61^ present vaccination-associated myo/pericarditis as rare, (typically) ‘mild’ and followed by rapid recovery with anti-inflammatory treatment. The reviews have not framed vaccine-associated risks versus infection-associated risks using compatible denominators based on exposure (vaccination) and infection (seroprevalence), thus the infection-associated risks may have been overstated by at least a factor of 4 according to CDC estimates of the burden of COVID-19 illness.^62^ However, vaccine-associated myocarditis has been found to occur in as many as 1 in 2652 males aged 12–17 years and 1 in 1862 males aged 18–24 years after the second dose^59^ (and as high as 1/1300 after the second dose in a BNT162b2–mRNA-1273 combination).^63^ An Israeli study described one in five cases among 16–29 year-olds to be of intermediate severity, meaning these cases had persistent new/worsening abnormalities in left ventricular function, or persistent ECG anomalies, or frequent non-sustained ventricular arrhythmias without syncope.^64^ The CDC reported that 1200 of the 1314 verified myocarditis cases with known hospitalisation status following the primary series or booster had been hospitalised.^65^ Among adolescents, 69%^66^–80%^67^ of those diagnosed with vaccine-associated myo/pericarditis had findings consistent with cardiac inflammation on MRI testing 3–8 months after the second dose. The potential long-term impact of scar tissue on heart conduction remains unknown.^66 67^ Postvaccination myocarditis has been found to be equivalent to or exceed the risk of post-COVID myocarditis in males less than 40 years old despite the lack of seroprevalence-based estimates of COVID-associated myocarditis.^68^ Rare incidences of death in young males attributed to mRNA vaccine-induced myocarditis have also been reported.^69 70^

### Limitations of analysis

These estimates have a number of limitations. First, our estimates rely on sponsor-reported and CDC summaries of AEs; we cannot account for failures to report small sample sizes, poor quality evidence subject to serious bias or loss to follow-up during the clinical trials. Second, our SAE estimate does not distinguish between specific types or the clinical significance of SAEs because of scarce data. The BNT162b2 RCT found more SAEs in the placebo group (24/5020) than the booster group (16/5055). However, blinded investigators attributed as vaccine-related three SAEs in the vaccine group (moderate persistent tachycardia, moderate transient elevated hepatic enzymes and mild elevated hepatic enzymes) and two SAEs in the placebo group (myocardial infarction and chest pain of unknown origin).^19^ Per million doses, the SAEs were therefore 593.5/million in the vaccine group vs 398.4/million in the placebo group, resulting in a risk difference of 195.1/million doses. The phase II/III BNT162b2 booster trial participants were of median age 42.0 and the company’s adolescent booster trial, for example, included only 78 individuals aged 16–17 years randomised to receive booster or placebo.^71^ Nevertheless, one male in this age group was hospitalised with myopericarditis after receiving a third dose of BNT162b2.^71^ It is possible that multiple severe side effects were reported by the same participant in the RCT trials and that the number of people impacted by such reactions is lower than our estimate. Hence, the causal relationship between our estimated SAEs and the COVID-19 vaccines needs to be approached with caution. We are extrapolating SAE data to young adults (18–29 years old) that were originally generated in clinical trials involving all age groups. However, studies have shown that younger people have a greater likelihood of vaccine-related AEs.^72^

More generally, data limitations affect the CDC’s ability to evaluate both BNT162b2 and mRNA-1273. For example, the CDC’s Grading of Recommendations, Assessment, Development, and Evaluation (GRADE) review^50^ noted ‘serious’ risk of bias for SAEs and ‘very low’ certainty of evidence (type 4) for all measures. While Pfizer conducted an RCT among 10 000 participants assigned 1:1 to booster or placebo, a sample size of 5000 is not sufficient to detect SAEs occurring at a rate of 1 in 7000 (such as vaccine-associated myo/pericarditis) among a subset of the population aged 18–29 years at highest risk. However, the trial data do suggest that the rate of AEs was higher among the intervention group than the placebo group (25.2% vs 6.8%).^73^ Moderna conducted a small, non-randomised safety study among 344 participants who elected to get a booster, and the reported SAEs were subject to serious risk of bias.^50^ ^4^

Despite these limitations, we believe that the data suggest caution is warranted. Haas *et al* 74 suggested that many systemic AEs in the RCTs (76% of systemic and 24% of local reactogenicity) may have been due to a nocebo effect—anxiety, expectations and background symptoms. It is very likely, however, that real-world severe or serious AEs may be greater than those reported in the RCT data because standard trials are underpowered to detect rare AEs and there may also be selection bias: those who had a reaction during the primary series may have a greater expectation of harmful side effects to the booster and are less likely to enrol in a trial. In fact, these data are usually collected after a drug has been approved and is on the market (phase IV clinical trial data). Such limitations show the need for more robust postmarketing data and ideally large, controlled trials to determine risks and benefits for any future booster doses, especially in younger age groups.

Universities have not published cumulative AE rates on their COVID-19 dashboards, thus there is no current way to validate these estimates with real-world data. Even with the residual uncertainties, our risk-benefit assessment shows that it is at least plausible that expected individual harms outweigh benefits for young healthy people (ie, most young adults), and it is implausible that individual benefits significantly outweigh risks. Pfizer’s own booster data support this inference.^71^ In requesting the EUA for boosting adolescent males, the BNT162b2 risk-benefit analysis estimated 23–69 cases of myocarditis per 1 million booster doses administered and 29–69 COVID-19 hospitalisations averted,^71^ yet this estimate of 23–69 cases of myocarditis per million third BNT162b2 doses administered is now known to be an order of magnitude below the 200.3 per million reported by the US CDC among adolescents aged 16–17 years.^51^ Finally, our NNV with a booster dose to prevent one hospitalisation likely errs on the side of overestimating the effectiveness of the booster. We do not incorporate the protective effects of prior infection, for example. Recent studies have found rapid waning of effectiveness against hospitalisation during Omicron to <50% by 3–4 months,^41^ with some studies failing to detect any significant benefit against hospitalisation of a booster dose among those <40.^21^ If accurate, these data would render our booster risk-benefit analysis even less favourable.

## Five ethical arguments against university booster mandates

Below, we present five ethical arguments against university booster mandates informed by our risk-benefit assessment and ethical analysis of mandatory policies to date. These arguments relate to (1) the importance of transparent, peer-reviewed risk-benefit analyses in policy, (2) the potential for net individual harm, (3) the lack of a proportionate public health benefit, (4) the lack of reciprocity in terms of compensation for vaccine-related harms and (5) the wider social harms of vaccine mandates.

### Transparency

Risk-benefit assessment is essential to the ethical acceptability of public health policy, and transparent, peer-reviewed assessments help maintain trust in public health, especially in the context of controversial policies. There is an even stronger rationale for thorough and transparent risk-benefit assessment when interventions are mandated or when (given uncertainty or relevant population differences) some people might face harms not outweighed by individual benefits. In such cases, risk-benefit assessments should be stratified by demographic factors and updated as new data become available to reduce uncertainty. At a minimum, if an intervention is implemented despite significant uncertainty (especially if it is mandated), there is a strong ethical rationale to collect (controlled) data to resolve relevant uncertainties.

An Omicron-era risk-benefit assessment published by the CDC and FDA could provide additional insight into the appropriateness of university booster mandates. However, such a risk-benefit assessment has not been published to date. Without such a formal analysis, professional associations (such as the ACC expert panel^61^) have been forced to infer from the literature and CDC’s own analyses. For example, the ACC expert panel produced a graphic displaying a favourable harms versus benefits ratio for the second dose among young adults aged 12–29 years.^61^ The ACC’s widely promoted graphic is tied to data presented by the CDC^75^ and relies on four key assumptions which bias the findings in favour of vaccination: (1) vaccine effectiveness of 95% over 120 days to prevent COVID-19 cases and hospitalisations; (2) myocarditis rates were derived from passive surveillance in VAERS instead of active surveillance available to the CDC (VSD) resulting in harms being underestimated by a factor of 10^51 52^; (3) harms and benefits were averaged across ages 12–29 when the risk may be highest among those aged 16–19 years^51 52^; and (4) hospitalisation rates were tied to May 2021 data, more than a year prior to the ACC’s review and pre-Omicron. Nevertheless, for adolescent males aged 12–17 years, the CDC estimated 56–69 myocarditis cases would be expected while 71 intensive care unit admissions could be averted.^75^

It was foreseeable that the decision to recommend boosters for all adults (against the advice of the FDA panel) would be followed by booster mandates since pandemic vaccine mandates were already in place in many universities and colleges throughout the USA at the time.^13^ Universities rely on public health agencies such as the CDC for guidance. Thus, we maintain that if mandates remain then it is critically important to update public risk-benefit estimates for boosters among adults younger than 40, stratified by sex, comorbidity status and history of infection to provide evidence that the intervention confers an expected net benefit to younger individuals in the context of the prevailing SARS-CoV-2 variants and pre-existing immunity. Without this, it is problematic to repeatedly and emphatically claim that COVID-19 vaccines are ‘safe and effective’ without specific risk-benefit analyses for different age categories and with consideration for individual health status, including evidence of prior infection, because risks of both disease and vaccination are highly variable according to these factors.^9 10^

Since there has not been any RCT specific to evaluating boosters in young adults, the CDC relied on data from an older cohort with a median age of 42.0–51.7^71 73^ and incorrectly assumed that the benefits would also outweigh risks for younger age groups. As we have shown, it is likely that this assumption is incorrect. Under such uncertainties, ethical vaccine policymaking arguably requires transparency about scientific knowledge and uncertainties regarding vaccine risks and benefits (ie, even more transparency than where certainty is high), and at the very least allows for shared decision-making aligned with an appreciation of stratified risks instead of placing the emphasis on simplistic messaging.

Transparent policymaking can encounter a ‘trust paradox’ in providing information about vaccine risks to the public. As noted by Petersen *et al*,^76^ governments have a perverse incentive to withhold negative information about vaccines since they are actively promoting such products and negative information about vaccines reduces vaccination uptake. And yet transparent disclosure about negative information (eg, side effects) helps sustain trust in health officials and reduces the politicisation of vaccines.^77^ Transparency may reduce the uptake of vaccination in the short term but will uphold trust in health authorities and vaccines in the longer term—just as open disclosure regarding clinical harms promotes trust in medicine.^78^ To address the ‘trust paradox’ in regulatory politics, and to maintain trust in government and scientific institutions, greater data accountability (in this case, a risk-benefit analysis) should precede any policy debate about mandates. Given concerns about pharmaceutical influence on the political process^78 79^ this should be facilitated by mechanisms to ensure independent scrutiny of regulatory science.^79^

### Potential net expected individual harm

The reasonable possibility of a net harm to individuals (as presented in our risk-benefit assessment) should provide a strong basis to argue for the ethical case against booster mandates for young adults. Mandates at institutions of higher education serve the age group with one of the lowest public health burdens from COVID-19. Hence, boosters provide a low and transient impact on transmission and hospitalisation for an age group with a vague and unquantified prospect of benefit. Arguably, this has been considered by most universities and colleges and is the reason why most do *not* have booster mandates for the fall of 2022. In fact, this is also likely why European countries, including the UK, France, Germany, Norway, Sweden and Denmark (to our knowledge), never had university-implemented mandates.^14^ When the European Centre for Disease Prevention and Control (ECDC), a body serving some 300 million European residents, recommended boosters in November 2021, priority was focused on those over age 40.^80^ Taking a different view of the data, in the fall of 2021 the US CDC recommended boosters for all adults and recommended a *second* booster for all Americans aged 50 years or more for fall 2022.^81^ The ECDC, in contrast, recommended that first boosters be ‘offered’ with prioritisation for those over 40 years, and now recommends second boosters for the 2022 autumn campaign only for those over age 60 and those with an immunocompromised status or high-risk medical conditions.^82^

Reflecting again to fall of 2021, the UK’s JCVI provides an example of using the potential for net harm to advise *against* the primary vaccination series for 12–15 year-olds.^83^ The JCVI argued that the potential benefit of vaccination in this age group was only ‘marginally greater than the potential known harms’, since healthy 12–15 year-olds are at very low risk of serious outcomes from COVID-19. Although it may be the case that the JCVI adopted worst-case estimates,^84^ such an approach reinforces the need to act judiciously under conditions of uncertainty where the clear benefits of an intervention are not confidently above the potential harms. Note also that they mention ‘potential known harms’ without taking into consideration potential long-term effects. The UK Health Ministers subsequently voted to offer a single dose of vaccination to adolescents aged 12–15 years in consideration of: ‘…the health and wider social benefits to this cohort’.^85^ A second dose was offered to those with underlying health conditions. There are important parallels between the JCVI decision and the outcome of the FDA panel that recommended against universal booster recommendations for adults in the USA in the fall of 2021: in both cases, the US and UK governments disregarded these recommendations. A key ethical difference is that the UK has not implemented any COVID-19 vaccine mandates at schools or universities, and the mandate proposed for care home and healthcare workers was withdrawn.^86^

As noted above, blanket mandates ignore widely available critical data, such as the benefits of prior infection and data on adverse effects. These factors make an expected net harm now even more likely than when mandates began and make it more urgent to update COVID-19 vaccine policy. Policies for other vaccines have been updated following the accumulation of new data. For example, adult boosters for tetanus and diphtheria vaccines (though previously widely administered) have been shown to provide no benefit.^87^ Vaccines for influenza, dengue and rotavirus have been withdrawn or had strict limitations placed on their use in children due to unexpected harms.^88^ Adenovirus-vectored COVID-19 vaccines have been limited in their use due to thrombosis (especially in younger women).^89^ Uncertainties remain regarding mRNA vaccines, for example, related to their effects on menstruation and fertility,^90^ shingles^91^ or the overall safety of current formulations in younger adults and children as well as evidence in support of booster vaccination.^92^

There are two other theoretical problems that could be factored into mandatory programmes from a precautionary standpoint: original antigenic sin and the non-specific effects of vaccines. Original antigenic sin refers to the decreased ability of an individual to respond to a new viral variant because the immune system has been ‘locked’ onto the original immunogen.^93^ While data have not shown this to occur with COVID-19 vaccine, it cannot yet be ruled out as an important side effect of repeat vaccination, including with the new bivalent booster. Non-specific effects of vaccination refer to the effects of a vaccine on overall health and all-cause mortality, which have been shown to differ based on the type of vaccine (eg, live vs non-live) and age/sex.^94 95^ Both of these theoretical issues are at the frontiers of our current knowledge of vaccinology and are rarely considered in the media and by the lay public. We cite these examples to support our main point: proportionality of mandates should account for uncertainty regarding evidence that benefits outweigh harms, especially as the marginal benefits of vaccination and boosting for young adults become vanishingly small with increased population immunity.

### Lack of proportionate public health benefit

Proportionality, a key principle in public health ethics, requires that the benefits of a public health policy must be expected to outweigh harms, including harms arising from the restriction of individual liberty and basic human rights such as access to education and employment.^1 5–8 86^ Where mass vaccination involves harm to a minority of individuals, or coercion or undue inducements are used to increase vaccine uptake, proportionality requires that these considerations be outweighed by public health benefits, typically in the form of reduced transmission from vaccinated individuals to others.^96^

COVID-19 booster mandates often involve a degree of coercion, including the threat of loss of access to education and free choice of occupation, disproportionately affecting disenfranchised groups.^96^ Contrary to those who restrict the concept of coercion to situations of a direct threat to something people should have access to as a matter of right,^97^ we endorse here a broader concept of coercion that includes situations of structural pressure that deprive people of reasonable options.^98^ ^99^ To be ethically acceptable, such severe restrictions of individual liberty need to be justified by an individual benefit and by the expectation that vaccination reduces harm to others. Booster doses of COVID-19 vaccines provide limited lasting reduction in the probability of infection or transmission,^27–29^ hospitalisation^41^ and limited expected benefits to young healthy individuals, especially those who have already been infected.^31–33 100–102^ The net expected harms to individuals and the harms of coercive mandates themselves are not counterbalanced by a large public health benefit (and in fact may harm the public health through the attrition of healthcare workers); such harms and restrictions of liberty are therefore disproportionate and ethically unjustifiable.

### Failure of reciprocity

The use of booster mandates raises an additional ethical problem of *reciprocity* for institutions of higher education and public health authorities.^103 104^ Most vaccines are covered in the USA^105^ and Canada^106^ by an injury compensation programme based on fair (reciprocal) compensation for those who experience a vaccine-related harm. Mandatory vaccines arguably require even stronger protections for individuals who experience consequences that lead to permanent harm^107^ because their free choice regarding vaccination has been limited. While institutions of higher education are mandating boosters, the US and Canadian compensation programmes have failed to uphold their social justice responsibility to injured individuals. In the USA, COVID-19 vaccines and therapeutics are processed by the Countermeasures Injury Compensation Program (CICP) which is designed to cover epidemics, pandemics and security threats as designated by the Secretary of Health and Human Services and as authorised by the Public Readiness and Emergency Preparedness (PREP) Act.^105^ As of 1 August 2022, thirty-seven claims have been denied compensation because ‘the standard of proof for causation was not met’ or ‘a covered injury was not sustained’.^108^ No claims have been paid out by the US CICP but one claim for anaphylaxis has been approved for compensation and payout is currently pending assessment of eligible expenses.^108^

The federal US vaccine injury programme has failed to compensate but one COVID-19 vaccine-injured individual in the context of booster mandates in place at hundreds of US universities.^108^ It is also important to note that boosters have been granted an EUA by the FDA, but are still not fully approved.^109^ Thus, universities and colleges that mandate COVID-19 boosters are pressuring young adults to receive a vaccine that, in case of injury, has no transparent legal route to adequate compensation. In sum, one core precondition for vaccine mandates is a functioning and fair compensation programme, which has not been achieved for COVID-19 vaccines.

### Wider social harms

Strong coercion may create significant social harms. COVID-19 vaccine mandates have generally involved a high degree of coercion, effectively ostracising unvaccinated individuals from society. University mandates involve significant coercion in that they exclude unvaccinated people from the benefits of university education (or employment) and thereby entail major infringements to free choice of occupation and freedom of association. When such mandates are not supported by a *compelling* public health justification and where exemptions are not easily available, the likelihood of reactance and negative social effects are increased.^1^ The social harms of university COVID-19 mandates have not been formally studied, but there is reason to think that they will be significant.^1^ Policies can have wide-ranging consequences for non-compliance, such as loss of employment, loss of internet use, restriction to off-campus versus on-campus housing, delays or refusal to process student housing requests, loss of enrolment, a hold placed on grades, inability to use recreational facilities to train or compete in sports, access to scholarships for competitive sports, registration for class and delays in ability to repay student loans after graduation. A number of young adults and professors affected by mandates have outlined publicly their perspectives and the social harms of these policies, such as loss of access to schooling and social services,^110^ psychosocial stress, reputational damage and lost income and threats of being disenrolled or deported.^111^ This punitive public health approach may also provoke reactance in young adults,^1^ with long-term negative consequences on trust in society and institutions and vaccine confidence in general, including vaccine hesitancy for routine paediatric and adult vaccines, a problem which predated the pandemic and is considered one of the WHO’s top 10 threats to global health.^112^

## Objections: possible rationales for mandates

Despite the considerations above, proponents of university COVID-19 booster mandates might argue that such policies are justified (even if some individuals experience uncompensated harms) because they: (1) help *normalise* compliance with vaccination as a social duty (thereby promoting solidarity or provaccine attitudes that undermine antivaccination sentiment) and/or (2) help to increase the safety of the university environment or wider society. Mandates may help some people ‘feel better’, knowing that everyone in a crowd, dorm or classroom is vaccinated, that they are among peers who have ‘done the right thing’ and ‘care about the safety of others’. For instance, some faculty and staff may ‘feel protected’ by the new booster mandate introduced at Western University in Ontario, Canada, on 22 August 2022.^113^ From this perspective, if a majority of university policymakers (whether clinical advisory group members, administrators and/or professors) or students *believe* that vaccination should be socialised to promote solidarity, counteract antivaccination sentiment or create a safe environment, then such beliefs (and values) should guide policy.

However, even if many people hold such beliefs and even if such goals are laudable, policy must be predicated on methods and models which are open to public scrutiny. Risk-benefit assessments should remain objective and avoid the use of some people feeling better or safer to justify behavioural rules with sanctions for non-compliance in the absence of rational justification. While many vaccines do improve group safety by reducing transmission, the current generation of COVID-19 vaccines does not provide significant lasting effects of this kind, and repeated doses appear to provide diminishing benefits (in terms of reduced infection) per dose, especially among young adults.^114^ It therefore makes little sense to claim, as a matter of policy, that COVID-19 vaccination is a prosocial act or that the unvaccinated are a disproportionate threat to others. Moreover, it is unclear whether *mandating* COVID-19 boosters will produce a net positive effect on provaccine sentiment in society—in fact, booster mandates may increase antivaccination beliefs and reduced uptake of other (non-coronavirus) vaccines.^1 86 96^ As highlighted above, there are also wider social harms of policies that purport to reduce transmission of a ubiquitous virus: such policies may create a fear of infection among young healthy people (out of proportion to the actual risks) and contribute to worsening mental health, an issue which predates the pandemic.^115^

Moreover, the claim that the *socialisation* of compliance with public health measures can justify those measures is problematic for three other reasons. First, such an argument is circular: compliance *should not be* an end itself; policy must be justified by the expectation of public health benefit. Second, people have different attitudes to compliance depending on their values (eg, the views regarding the importance of individual liberty) and experiences (eg, those with low baseline levels of trust in public health due to negative experiences of health professionals or government agencies). Policies that require people to comply against their values and preferences require ethical justification, especially where voluntary compliance is likely to be lower among those who are disempowered (eg, students) or marginalised for other reasons,^5 116^ for example, those from social groups which have been mistreated by government agencies or by the medical system in the past, including in the context of research.^117^ Third, the socialisation argument is based, in part, on concepts of civic duty and responsibility to others. Pushing for boosters even when these will not significantly contribute to overall risk reduction runs counter to the responsible use of public resources. Policies that encourage waste of valuable healthcare resources, to make some feel better, are sending a distorted message about important societal obligations.

The proclivity for university vaccine mandates may also reflect harmful trends towards intolerance in university bureaucracies that value compliance over individual freedoms. Mandates, by their nature, encourage conformity and acquiescence to authority, and exclude those with different views or values. Though universities might take pride in being places that permit the free exchange of ideas, mandates reduce the scope for reasoned debate regarding scientific uncertainties or conflicts of ethical values.^118^ For example, how many universities have held public debates about mandatory COVID-19 vaccination? To our knowledge, very few such debates have taken place in North American institutions. We are aware of only one academic event^119^ which some of us organised, in which mandates were critically debated. Sanctions for lack of full vaccination imposed on university professors who publicly voiced their opposition against mandates could arguably also have been intended to suppress public debate or be interpreted as such.

## Implications for broader COVID-19 vaccine mandates for youth in schools and other institutions

The arguments presented above are relevant to third, fourth or fifth-dose booster mandates and to university or school policies that maintain primary two-dose COVID-19 vaccine mandates in 2022 in the face of high rates of previous SARS-CoV-2 infection.^13^ Two-dose mandates are being upheld in at least 1000 universities and colleges across the USA, far more than the 300 or so maintaining booster mandates,^2^ and also some primary and secondary schools in the nation’s largest public school systems^120^ which instituted mandates then extended the deadline for compliance when it was apparent that serious inequities in access to education would result.^121^ It is even harder to justify a two-dose primary vaccine mandate in late 2022 than when such policies began in mid-2021.^46^ This rationale is weak at best and wrong at worst. Consistent with our argument above, the now high prevalence of prior infection, data regarding the lack of sustained transmission reduction by current vaccines and the age at peak risk for myo/pericarditis being young adults aged 16–17 years^51^ all undermine the case for two-dose vaccine mandates. Students heading to colleges with mandates must currently upload proof of vaccination in order to enrol or be assigned to on-campus housing. We would therefore urge universities and schools to rescind all COVID-19 vaccine mandates. Strong statements in support of mandates made in 2021 by organisations such as the Association of Bioethics Program Directors in North America,^122^ the American Civil Liberties Union^123^ and the Ontario Human Rights Commission^124^ are now obsolete. Such organisations have an ethical obligation to revise these public statements and consider whether they are valid in light of current data.

The continued policy of two-dose mandates may represent *status quo* bias: when indiscriminate regulations are normalised they often remain even when it has no (current) rational basis. The more rules, the more paperwork and cumbersome ‘busy work’ administrators and young students and professionals need to complete. Yet rules come with consequences: how much are universities, corporations, consulting firms and the military paying in staff time to monitor and maintain vaccine mandates? How much time and energy are young adults using to comply with these policies? How much frustration and psychosocial stress is this causing? What are the consequences of attrition of healthcare workers and military service members at times when the labour market is tight and recruitment is difficult? When vaccine mandates are unethical, individuals may have an ethical duty to oppose them, in part to promote tolerance and prevent further bureaucratic encroachment and disenfranchisement of individuals with reasoned arguments against such mandates. Finally, we argue that institutions have an ethical duty to evaluate the effectiveness of such programmes if the status quo is to be maintained.

## Conclusion

Based on public data provided by the CDC,^19^ we estimate that in the fall of 2022 at least 31 207–42 836 young adults aged 18–29 years must be boosted with an mRNA vaccine to prevent one Omicron-related COVID-19 hospitalisation over 6 months. Given the fact that this estimate does not take into account the protection conferred by prior infection or a risk adjustment for comorbidity status, this should be considered a conservative and optimistic assessment of benefit. Our estimate shows that university COVID-19 vaccine mandates are likely to cause net expected harms to young healthy adults—for each hospitalisation averted we estimate approximately 18.5 SAEs and 1430–4626 disruptions of daily activities—that is not outweighed by a proportionate public health benefit. Serious COVID-19 vaccine-associated harms are not adequately compensated for by current US vaccine injury systems. As such, these severe infringements of individual liberty and human rights are ethically unjustifiable.

Mandates are also associated with wider social harms. The fact that such policies were implemented despite controversy among experts and without updating the sole publicly available risk-benefit analysis^19^ to the current Omicron variants nor submitting the methods to public scrutiny suggests a profound lack of transparency in scientific and regulatory policy making. These findings have implications for mandates in other settings such as schools, corporations, healthcare systems and the military. Policymakers should repeal COVID-19 vaccine mandates for young adults immediately and ensure pathways to compensation to those who have suffered negative consequences from these policies. Regulatory agencies should facilitate independent scientific analysis through open access to participant-level clinical trial data to allow risk-stratified and age-stratified risk-benefit analyses of any new vaccines prior to issuing recommendations.^125^ This is needed to begin what will be a long process of rebuilding trust in public health.

## Data Availability

All data relevant to the study are included in the article or uploaded as supplementary information. The data are cited in table 1 and in the references. All data and calculations are included in the manuscript. We are providing the following citations as well: 18. Oliver S. Updates to the evidence to recommendation framework: Pfizer-BioNTech and Moderna COVID-19 vaccine booster doses. ACIP Meeting. 19 November 2021 (Slides 26, 29, 30, 31, 37). Available at: https://www.cdc.gov/vaccines/acip/meetings/downloads/slides-2021-11-19/06-COVID-Oliver-508.pdf. Accessed on 28 March 2022; 50. CDC. Grading of Recommendations, Assessment, Development, and Evaluation (GRADE): Pfizer-BioNTech, Moderna, and Janssen COVID-19 booster doses. 29 October 2021. Available at: https://www.cdc.gov/vaccines/acip/recs/grade/covid-19-booster-doses.html%23table-03a; 51. Shimabukuro T. Update on myocarditis following mRNA COVID-19 vaccination. Advisory Committee on Immunization Practices (ACIP). 23 June 2022. Available at: Update on myocarditis following mRNA COVID-19 vaccination (cdc.gov). Slides 10 and 23. Accessed on 20 August 2022; 52. Shimabukuro T. Myocarditis following mRNA COVID-19 vaccination. Advisory Committee on Immunization Practices (ACIP). 19 July 2022. Available at: Myocarditis following mRNA COVID-19 vaccination (cdc.gov). Slides 11 and 23. Accessed on 20 August 2022; 53. Sharff KA, Dancoes DM, Longueil JL, *et al*. Myopericarditis after COVID-19 booster dose vaccination. *Am J Card* 2022;172:165–166. https://doi.org/10.1016/j.amjcard.2022.02.039; 54. Friedensohn L, Levin D, Fadlon-Derai M, *et al*. Myocarditis following a third BNT162b2 vaccination dose in military recruits in Israel. *JAMA* Apr 26;327(16):1611–1612. doi:10.1001/jama.2022.4425.

## Footnotes

Offit recommended that his own son not receive a booster dose due to concerns that benefits would not outweigh risks (see: https://www.theatlantic.com/health/archive/2022/01/should-teens-get-booster-omicron/621222/).

See also: https://www.fda.gov/safety/reporting-serious-problems-fda/what-serious-adverse-event (accessed 20 October 2022).

Table 3e footnote h: Overall, 4/344 (1.2%) participants experienced five SAEs during a median follow-up of 5.7 months after booster dose (administered at least 6 months after a 50 μg (n=173) or 100 μg (n=171) two-dose primary series); the sponsor deemed these unrelated to mRNA-1273. Data on an equivalent primary series comparison group were not available at the time of the GRADE assessment.

Table 3e, footnotes (h), (i), (j): h. Overall, 4/344 (1.2%) participants experienced five SAEs during a median follow-up of 5.7 months after booster dose (administered at least 6 months after a 50 μg (n=173) or 100 μg (n=171) two-dose primary series); the sponsor deemed these unrelated to mRNA-1273. Data on an equivalent primary series comparison group were not available at the time of the GRADE assessment. i. Comparator group is 100 µg primary series recipients in the phase II randomised dose confirmation study. j. Participants’ ability to choose whether to receive a booster likely introduced selection bias (eg, those with adverse events or reactions with the primary series may have been less likely to choose a booster).
